# Linear Accuracy of Intraoral Scanners for Full-Arch Impressions of Implant-Supported Prostheses: A Systematic Review and Meta-Analysis

**DOI:** 10.1055/s-0042-1758798

**Published:** 2023-01-30

**Authors:** Franciele Floriani, Guilherme Carpena Lopes, Alexandre Cabrera, Wagner Duarte, Panagiotis Zoidis, Dayane Oliveira, Mateus Garcia Rocha

**Affiliations:** 1 Department of Periodontology, University of Florida College of Dentistry, Gainesville, Florida, United States; 2 Universidade Federal de Santa Catarina, Florianópolis, SC, Brazil; 3 Department of Dentistry, Federal University of Santa Catarina, Florianópolis, SC, Brazil; 4 Division of Prosthodontics, Department of Restorative Dental Science, University of Florida, Gainesville, Florida, United States; 5 Department of Periodontology, University of Florida, Gainesville, Florida, United States; 6 Department of Restorative Dental Sciences, Center for Dental Biomaterials, College of Dentistry, Gainesville, Florida, United States

**Keywords:** systematic review, meta-analysis, digital dentistry, implant-supported dentures, intraoral scanners, impression materials

## Abstract

This article compares the accuracy of intraoral scanners (IOSs) used in the digital impression of full arches to fabricate implant-supported complete prostheses. This study followed the Preferred Reporting Items for Systematic Reviews and Meta-Analyses guidelines and was registered in the Open Science Framework (DOI 10.17605/OSF.IO/CPM9K). Six electronic databases, gray literature databases, and a manual search were performed in April 2022. Studies that evaluated the accuracy of intraoral scan impressions compared with conventional impressions in full-arch impressions were included for complete implant-supported prostheses. In addition, an adapted checklist for reporting in vitro studies was used to assess the risk of bias. Meta-analysis was conducted using a random-effects Hunter– Schmidt model. Nine studies were included in the analysis. IOS impressions present higher accuracy (137.86 μm) than conventional impressions (182.51 μm) ( *p* <0.001). The heterogeneity of the study's methodology was I2¼18.34. However, impression accuracy varies significantly with scan body type, IOS type, scanning strategy, and modification technique. For most IOS systems, the acceptable clinical threshold of linear accuracy of 200 μm can be achieved, except for the True Definition Scanner in one of the studies. Based on the results of the included studies, digital impressions using IOS present similar or better linear accuracy than conventional impression techniques.

## Introduction

The accuracy of dental impression is essential for the predictability of oral rehabilitation treatment. 1 2 That is influenced by several factors, type of tray (stock vs. custom), 3 4 quality of the impression material, compatibility with the impression material, and gypsum pouring technique. 5 Additionally, the accuracy of implant impression techniques can be influenced by the angulation of the implants, the type of implant-abutment connection, and splinting material. 6 7 However, some of these variables can be eliminated with the advent of digital impressions. 8 9

There are two different types of digital scanners for dental impressions: intraoral scanners (IOSs) and benchtop scanners. 10 11 IOS exhibits advantages such as fewer materials (no need for trays, adhesives, and dispensers), better control of the aseptic chain (tip can be sterilized), ease of duplication, and easy storage (there is no expiration date), and recoverability of files. 5 12 Several studies have been published assessing the accuracy of digital impressions for single unit restorations 13 14 and quadrants. 15 16

For instance, Flügge et al evaluated the marginal accuracy of crowns employing digital impression techniques. As a result, the digital impression showed comparable results to the conventional impression. Likewise, studies comparing conventional and digital implants impression have demonstrated similar results between the techniques. 17 18

On the other hand, the use of IOS has shown limitations for full-arch cases. These limitations are related to the accuracy of the scanning strategy. 19 20 Several *in vitro* studies are comparing the accuracy of IOS for full-arch implant impressions; however, there are no systematic reviews of these studies to identify if IOS can perform similar to conventional impressions in full-arch implant impressions. A much-debated question is whether the current scientific evidence of *in vitro* studies can determine if IOS is more accurate than impression materials for the impression of complete implant-supported prostheses. Therefore, the aim of this study was to perform a systematic review and meta-analysis to answer the following question: What is the accuracy of IOS compared with conventional full-arch impressions used in the fabrication of implant-supported prostheses? The research hypothesis of this study was that there would be no significant differences in the accuracy of impressions made with IOS or conventional impression materials.

## Methods

This systematic review followed the Preferred Reporting Items for Systematic Reviews and Meta-Analyses, 21 and was registered at the Open Science Framework under the DOI 10.17605/OSF.IO/SH3J2.

**Fig. 1:**
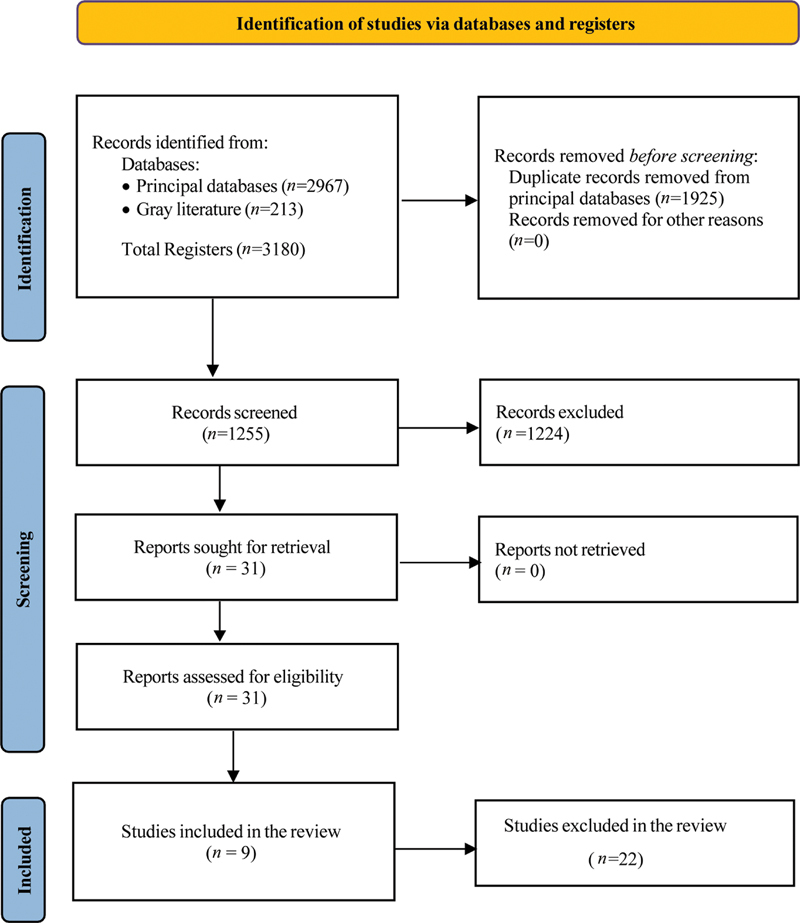
Flowchart of the identification, screening, and inclusion of the studies.

The focused question was structured using the PICO acronym. Population (P) were models with fully edentulous arches, intervention (I) was impressions with IOSs, comparison (C) was conventional impressions with impression materials, outcomes (O) were linear and angular deviations, and the type of study (S) was comparative *in vitro* experimental studies. To answer the focused question, inclusion criteria consisted *in vitro* study that compared conventional and IOS impressions in full arches for implant-supported prostheses. Papers written in any Roman Latin language were included. Conversely, observational studies, studies that evaluated partial arches, that did not compare digital impressions (intraoral) and conventional impressions, as well as reviews, letters, abstracts, and case reports were excluded. The main search strategy was formulated and applied in the PubMed (MEDLINE) database. Furthermore, the main search strategy was used as a reference and applied to the following databases: Scopus, Web of Science, LILACS, Cochrane Library, and EMBASE in April 2022. Additionally, a gray literature search was performed on Google Scholar, Clinical Trials, Open Gray, and ProQuest. Besides, a manual search on the Internet and in references within the articles was performed ( Supplementary Table S1 ). Two independent reviewers participated from the first phase of the study in the article's selection based on the title and abstracts' information using online software for systematic reviews (Rayyan, Qatar Computing Research Institute). After that, the intra- and inter-examiners calibration level was performed with the first 10% of the references. The acceptable level of agreement (> 7.0) among reviewers was achieved, and the second phase of the study (full-text) was performed ( Fig. 1 ).

**Fig. 2:**
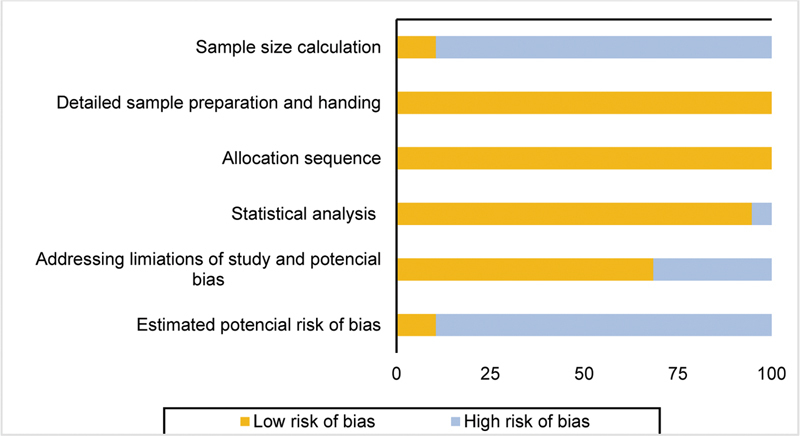
Qualitative analysis with adapted checklist for reporting *in vitro* 25

Data extraction was performed independently by two reviewers for the primary outcomes and compared collected data. The following data were extracted from the eligible studies: the purpose of the study, study details, sample features, scanning methods, measurement details, findings, and conclusion ( Table 1 ). Two independent and blinded reviewers performed the risk of bias using an adapted Checklist for Reporting *In vitro* Studies tool. 22 The tool comprised questions related to five domains: sample size calculation, sample preparation and handling, blinding, statistical analysis, and limitations and potential bias of the study. Each question was scored with “yes,” “no,” or “unclear.” Studies that fulfilled all questions were determined to be high-quality studies (low risk of bias), whereas studies containing 3 or 4 “yes” scores were considered medium-quality articles (moderate risk of bias) ( Table 2 ). Furthermore, studies with fewer than 3 “yes” scores presented low methodological quality (high risk of bias). The analysis was performed using a software program (RevMan 5.4; The Nordic Cochrane Center) ( Fig. 2 ).

**Table 1:** Characteristics of each included study

			Groups					
			Control group (convention impression)		Experimental Group (digital impression)			
Study, year Country	*N* total (master model material)	Jaw status	Material used Technique used Pouring material	Measurement ( *n* )	Scanner used ( *n* = total scannings) Scanning technique	Measurement	Main conclusions
Abdel-Azim et al, 2014 USA	1 (NR)	Edentulous mandible and maxilla	Polyether (Impregum, 3M ESPE, USA) ClosedTray Type IV Stone (Resin Rock, Whip Mix)	Image Pro version 6.2.1.491 (Media Cybernetics, USA) ( *n* = 2)	iTero (Cadent) ( *n* = 6) Unclear	Infrastructure level (Marginal discrepancy was assessed with the stereomicroscope at two points for each retainer on each model, buccally and lingually)	For complete-arch frameworks, the digital impression/fabrication technique resulted in an overall more accurate fit when compared with the conventional impression/fabrication method
Albaryak et al, 2021 USA	1 (Polyurethane)	Edentulous mandible	Polyvinyl siloxane, Elite HD (Zhermack SpA, Italy) OpenTray Type IV Stone (Fujirock, Japan)	Unclear	Carestream 3500, Cerec Omnicam; 3Shape Trios ( *n* = 30) Right posterior region and continued toward left posterior region on the opposite side of the arch and the scan paths were determined according to the instructions	STL level (The reference points were determined on the scan bodies. Two circles were created at 0.7 and 3.4 mm from the triangular pyramid base to center the upper and lower parts of the scan bodies. The center points of these circles where the distance measurements)	Complete arch implant case with high angulations and asymmetric distribution, digital impression methods achieved superior results in both distance and angular parameter than conventional method using nonsplinted open tray impression technique. Besides different acquisition methods and working principles of IOS can affect the accuracy
Alikhasi et al, 2018 Iran	2 (Acrylic resin)	Edentulous mandible and maxilla	Vinyl siloxanether (Zhermack Elite HD + Regular Body, Italy) Open tray/closed tray Type IV Stone (Herostone, Vigodent Inc., Brazil)	Coordinate-Measuring Machine (Mistral, DEA Brown&Shape, Italy) ( *n* = 3) Start to scan in the palatal of the right tuberosity and lingual surfaces of all scan bodies. Next, the buccal and then the occlusal	Trios (3Shape) ( *n* = 60) Unclear	STL level (Superposition of STL data sets [best-fit alignment tool])	Digital impression is better than the direct technique in the edentulous arch with straight and tilted implants, and both are more accurate than the indirect technique
Amin et al, 2017 USA	1 (Type IV Stone)	Edentulous mandible and maxilla	Impregum, 3M ESPE, USA) Open tray Type IV Stone (Resin Rock, Whip Mix)	Activity 880 scanner (Smart Optics, Germany) ( *n* = 1)	CEREC Omnicam True Definition ( *n* = 20) Started at the right retromolar. Continuous stroke was completed along the occlusal surface until the left retromolar and lastly buccal scan	STL level Superposition of STL data sets (best-fit alignment tool)	Omnicam was more accurate than conventional impressions (splinted open-tray technique). True Definition scanner had significantly less 3D deviations when compared with the Omnicam (precision)
Kim et al, 2019 South Korea	1 (Epoxy resin)	Edentulous maxilla	Vinyl siloxanether (Aquasil XLV; Dentsply Sirona); plastic tray: high-viscosity silicone impression material (Aquasil EasyMix Putty) Open tray Type IV dental stone (MG Crystal Rock, Maruishi)	Unclear	TRIOS 3 (3Shape, Denmark) ( *n* = 10) Scanning started from the occlusal at the right molar, continued to the scan body at the contralateral left first molar area, then to the palatal, and finally to the buccal	STL level (Superposition of STL data sets [best-fit alignment tool])	Intraoral digital scan resulted in less accurate trueness than the conventional open-tray impression technique in terms of overall linear displacement. The conventional open-tray impression technique resulted in more accurate precision for all the implant replica locations, and produced significantly smaller angular deviations compared with the intraoral digital scan
Menini et al, 2018 Italy	1 (Metal framework)	Edentulous maxilla	Polyether (Impregum, 3M ESPE, USA) Open tray/Closed tray		True Definition (3M ESPE) ( *n* = 35) Unclear	Infrastructure level (Marginal discrepancy was assessed with the microscope)	The use of an intraoral digitizer might represent a viable alternative to traditional impression materials for the fabrication of full-arch implant-supported prostheses provided with a satisfactory passive fit
Papaspyridakos et al, 2016 USA	1 (Acrylic resin)	Edentulous mandible	Group I: splinted coping impression technique at the implant level Group II: nonsplinted coping impression technique at the implant level Group IV: from the splinted coping impression technique at the abutment level		Trios (3Shape) Group III: from the digital impression technique at the implant level	One digital scan of the master cast at the implant level and one scan at the abutment level, with the same high-resolution extraoral scanner at 6 lumens precision (IScan D103i; Imet- ric), were used as control (golden reference)	The implant-level, splinted impressions were more accurate than the nonsplinted conventional impressions for completely edentulous patients • The accuracy of abutment-level, splinted impressions were not different than the nonsplinted impressions for completely edentulous patients • The accuracy of implant impressions is not affected by the implant angulation up to 15 degrees for completely edentulous patients. The connection type seems to affect accuracy because abutment-level impressions had no statistically significant differences from the control, whereas differences were identified for the implant-level, nonsplinted impressions. Seems to affect accuracy because abutment-level impressions had no statistically significant differences from the control
Rech-Ortega et al, 2019 Spain	1 (Titanium)	Edentulous mandible	Polyether (Impregum Penta Soft, 3M ESPE) Type IV plaster (Vel Polyether, Impregum Penta Soft, 3M ESPE) Type IV plaster (Vel MixStone, Kerr)/Type IV plaster (Vel- MixStone, Kerr)	Dental Designer, 3Shape ( *n* = 120)	True Definition (3M ESPE) Unclear	Six cylindrical scan bodies were screwed onto the analogues and the following distances were measured: between adjacent analogues from center to center: 1–2, 2–3, 3–4, 4–5, 5–6; between intermittently positioned analogs: 1–4, 3–6; between the most distal analogs: 1–6. This process was repeated for the 20 physical models. Five parameters (X, Y, Z, module XY, module XYZ) were calculated for each distance 1–2, 2–3, 3–4, 4–5, 5–6, 1–4, 3–6, 1–6 measured from the 20 physical models elastomeric impression material (EIM) and the master model	For adjacent analogs, the direct technique can be considered the most accurate for the XYZ module distance 1–2 as no statistically significant differences were found ( *p* = 0.146) in relation to the master model. For the other distances, 2–3, 3–4, 4–5, and 5–6, neither technique was completely accurate. True Definition (3M ESPE) (SDM) provided accurate data, without statistically significant differences in comparison with the master model ( *p* = 0.255)
Tan et al, 2019 Singapore	2 (PMMA)	Edentulous maxilla	Polyether (Impregum Duosoft, 3M ESPE, Germany)/Type IV dental stone models (Silky Rock, Whip Mix Co.)	Laboratory scanner (Straumann CARES Scan CS2 Visual 8.0 software, Institut Straumann AG, Basel, Switzerland) ( *n* = 6)	Trios 3Shape (3Shape) True Definition (3M ESPE) Unclear	The axial portion of each scan body was measured via eight virtual probe hits at two levels to define a cylinder (Trios, True Definition, inEos X5, and D900) or cone (Ceramill Map400)	Reducing interimplant distance may decrease global linear distortions (dR) for intraoral scanner systems but had no effect on Impregum and the dental laboratory scanner systems. Impregum consistently exhibited the best or second-best accuracy at all implant locations, while True Definition exhibited the poorest accuracy for all linear distortions in both models A and B. Impression systems could not be consistently ranked for absolute angular distortions

Abbreviations: 3D, three-dimensional; IOS, intraoral scanner; PMMA, polymethyl methacrylate; STL, stereolithography.

**Table 2:** Quality assessment and risk of bias of eligible studies with adapted Checklist for Reporting *In vitro* (CRIS guidelines) 22 tool

First author/Year	Sample size calculation	Detailed sample preparation and handing	Allocation sequence	Statistical analysis	Addressing limitations of study and potential bias	Estimated potential risk of bias
Abdel-Azim, 2014	N	Y	Y	Y	Y	Y
Alikhasi, 2018	N	Y	Y	Y	Y	Y
Albaryak, 2021	N	Y	Y	Y	Y	Y
Amin, 2017	N	Y	Y	Y	N	N
Menini, 2018	N	Y	Y	Y	Y	N
Papaspyridakos, 2016	N	Y	Y	Y	N	N
Kim, 2019	N	Y	Y	Y	N	N
Rech-Ortega, 2019	N	Y	Y	Y	Y	N
Tan, 2019	N	Y	Y	Y	N	N

The date for the linear accuracy of the nine included studies was obtained. The groups were divided based on the IOS system used in the study. The mean and standard deviation for each IOS reported in the study were used to perform the meta-analysis model. All data were imported to statistical analysis software (Stata/MP 17, StataCorp, College Station, Texas, United States). A random-effect Hunter–Schmidt model was used for the meta-analysis to compare the mean and standard deviation of each study's conventional and digital implant impression systems for each IOS at a significance level of *α* = 0.05.

## Results

The initial search resulted in 3,018 articles, which was reduced to 1,255 after removing duplicate reports. After study selection, 31 articles were selected for full-text reading, and 9 studies were considered eligible for this review in April 2022 ( Fig. 1 ). Because of the heterogeneity of the parametric data in the studies regarding the trueness, precision, and angular accuracy, the meta-analysis was performed only with linear accuracy. The reasons for exclusion can be found in Supplementary Table S2 .

The dimensional distortion of trueness and precision was described using deviation/discrepancy values. 21 This is done through a different methodology of IOS measurement technique, four studies used superposition of stereolithography (STL) data sets (best-fit alignment tool), which calculate the horizontal linear distance and the marginal discrepancy. Other studies analyzed the measurement technique through contact feature mode and marginal discrepancy evaluated with a microscope. Another intervention characteristic was the IOS measurement level executed on the infrastructure level, STL level, and cast level.

The majority of studies included only open tray conventional impressions, 1 20 23 24 except for two studies 4 24 which compared both closed tray and open tray to digital scans, one study used only close tray, 25 unclear in two studies, 2 26 and one study 15 evaluated splinted coping impression technique at the implant level, nonsplinted coping impression technique at the implant level, and splinted coping impression technique at the abutment level. Vinyl polysiloxane and polyether were used as impression materials for the conventional methods.

**Fig. 3:**
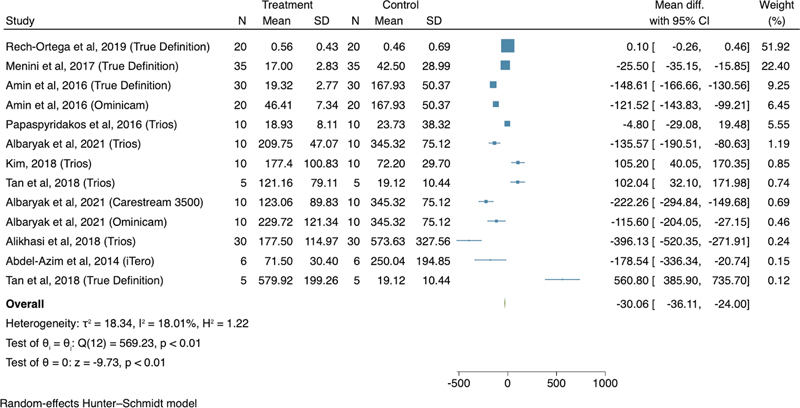
Forest plot for the accuracy of digital (treatment) and conventional (control) impressive techniques. The overall heterogeneity and p-values are based on a random-effects Hunter-Schmidt meta-analysis model with a level of significance of 0.05

The accuracy in conventional impression from *in vitro* ranged from 0.46 to 573.63 µm, and IOS impression ranged from 0.56 to 579.92 µm ( Table 3 ). Overall, the digital impressions present higher accuracy (137.86 µm) than conventional impressions (182.51 µm) ( *p* < 0.001; mean difference with 95% confidence interval of 30.06 µm [–36.11 to –24.00]). The heterogeneity of the study's meta-analysis was 18.01%, which suggests that the heterogeneity is insignificant, and the summarization of the meta-analysis results is statistically appropriate( Fig. 3 ). 27 Out of the nine studies that were included in the quantitative analysis, five studies 4 15 20 23 26 used Trios (3Shape, Copenhagen, Denmark), four studies 1 2 24 26 used True Definition (3M ESPE, St Paul, Minnesota, United States) IOS, two studies 1 20 used Cerec Omnicam (Dentsply Sirona, Bensheim, Germany), one study 20 used Carestream 3500 (Carestream Dental LLC, Atlanta, Georgia, United States), and one study 25 used iTERO (Align Technology, San Jose, California, United States).

**Table 3:** Outcomes of *in vitro* included studies

	Control group (conventional impression)		Experimental group (intraoral scanning)	
Author, year	Trueness distortion (mean ± SD)	Precision distortion (mean ± SD)	Trueness distortion (mean ± SD)	Precision distortion (mean ± SD)
Abdel-Azim et al, 2014	Linear: 250.04 ± 194.85 µm Angular: NR	Linear: NR Angular: NR	Linear: 3Shape Trios: 71.50 ± 30.40 µm Angular: NR	Linear: NR Angular: NR
Albaryak et al, 2021	Linear: 345.32 ± 75.12 μm Angular: 0.74° ± 0.65°	Linear: 66.97 ± 36.69 μm Angular: 0.50° ± 0.38°	Linear: Carestream: 123.06 ± 89.83 μm Cerec Omnicam: 229.72 ± 121.34 μm 3Shape Trios: 209.75 ± 47.07 Angular: Carestream: 0.26° ± 0.07° Cerec Omnicam: 0.53° ± 0.42° 3Shape Trios: 0.33° ± 0.30°	Linear: Carestream: 80.43 ± 29.69 μm Cerec Omnicam: 94.06 ± 69.96 μm 3Shape Trios: 35.55 ± 28.46 μm Angular: Carestream: 0.19° ± 0.11° Cerec Omnicam: 0.30° ± 0.28° 3Shape Trios: 0.22° ± 0.19°
Alikhasi et al, 2018	Linear: 573.62 µm Angular: 36.92°	Linear: NR Angular: NR	Linear: 3Shape Trios 177.50 µm Angular: 0.475°	Linear: NR Angular: NR
Amin et al, 2017	Linear: 167.93 ± 50.37 µm Angular: NR	Linear: NR Angular: NR	Linear: Omnicam: 46.41 ± 7.34 µm True Definition: 19.32 ± 2.77 µm Angular: NR	Linear: NR Angular: NR
Papaspyridakos et al, 2016	Linear: 110.58 µm Angular: NR	Linear: NR Angular: NR	Linear: 3Shape Trios: 19.38 µm Angular: NR	Linear: NR Angular: NR
Menini et al, 2018	Linear: 42.5 ± 28.99 µm Angular: 0.316 ± 0.167°	Linear: NR Angular: NR	Linear: True Definition: 17 ± 2.83 µm Angular: True Definition: 0.257° ± 0.242°	Linear: NR Angular: NR
Kim et al, 2019	Linear: 72.2 ± -µm Angular: 1.19 ± -° ^§^	Linear: 26.25 µm Angular: NR	Linear: 3Shape Trios: 177.4µm Angular: 3Shape Trios: 1.55°	Linear: 3Shape Trios: 80.76 µm
Rech-Ortega et al, 2019	Linear: 15.3 µm Angular: NR	Linear: NR Angular: NR	Linear: True Definition: 28.75 µm Angular: NR	Linear: NR Angular: NR
Tan et al, 2019	Linear: 0.10 μm Angular: 20.95	Linear: NR Angular: NR	Linear: –0.837 μm Angular: 288.34°	Linear: NR Angular: NR

Abbreviations: NR, not reported; SD, standard deviation.

For the Trios system, the mean accuracy difference was –48.62 µm (–131.39, 34.16) to the control group. Two studies 4 20 demonstrated better accuracy, and one study 15 showed no differences between Trios and conventional impressions. However, two studies 23 26 demonstrated lower accuracy for Trios when compared with conventional impressions. The mean accuracy difference for the True Definition system was –16.62 µm (–22.00, –11.24) to the control group. Two studies 1 24 demonstrated better accuracy, and one study 2 showed no differences between True Definition and conventional impressions. However, one study 26 demonstrated lower accuracy for True Definition when compared with conventional impressions. For Omnicam, the mean accuracy difference was –121.17 µm (–142.80, –99.53) to the control group. The two included studies 1 20 demonstrated better accuracy for the Omnicam than conventional impressions. For Carestream 3500 20 and iTero 25 the included studies showed better accuracy, and the accuracy mean differences were –222.26 µm (–294.84, –149.68) and –178.54 µm (–336.34, –20.74), respectively. The accuracy of 200 μm is reported as the maximum clinically acceptable discrepancy threshold. 28 All studies reported a mean accuracy difference from the control group below 200 μm, with the exception of the True Definition in the Tan et al 26 study.

## Discussion

This systematic review was based on nine *in vitro* studies. The data from these studies have an informative value for the clinician and provide substantial evidence on the different techniques for impression (conventional vs. digital) of full-arch impression for implant-supported prostheses. The research hypothesis of this study that there will be no significant differences in the accuracy of impressions made with IOS or conventional impression materials was rejected. In general, most IOS and conventional methods had trueness and precision values acceptable for clinical use. The studies showed that in conventional methods, the trueness and precision are influenced by impression material, impression technique, cast material, pouring technique, and measuring technique used by the researcher to evaluate the discrepancy. 5 12 13 The deviation values differed in digital methods according to the scanner system, scanning method, measurement technique, merging technique, scanning strategies, and learning curves of IOS. 29 30

The scanners are devices that digitize intraoral conditions in a three-dimensional (3D) file—these exhibit formats with three magnitudes that are important for becoming the digital impression valuable in dentistry. First, accuracy is defined (International Organization for Standardization 5725–1) in terms of trueness and precision. 31 The trueness is the deviation of the object scanned with an IOS from its real geometry; this means that accuracy is described by the mean of the discrepancies between the object scanned and the IOS scans of the object (target). Lastly, precision represents the discrepancies between the repeated scans of the same object performed with the same IOS and parameters. It represents how much a measurement could be systematically repeated. Precision is described by the mean of the discrepancies among the various scans of the object performed with the IOS. 32

In other words, the accuracy of digital (either intraoral or desktop) and conventional impressions was defined as the closeness agreement between a data set and the accepted reference value divided into the deviation between measured dimensions and actual dimensions of the object (trueness) and closeness of repeated measurements (precision). 32

According to Abdel-Azim et al, conventional impression showed a high linear trueness deviation value of 250.04 µm. The reason the conventional impression group was less trueness than the digital impression groups in this study may be associated with the nonsplinted open tray technique. Still, the Trios IOS accuracy of 71.50 ± 30.40 µm. Although there is not yet a scientific consensus, a value of 200 µm has been reported by the literature as acceptable in terms of prosthesis misfit. 33

In 2018, Alikhasi et al compared the trueness of the nonsplinted open tray and closed tray techniques using polyvinyl siloxane and the digital impressions made with the 3Shape Trios scanner. The impressions were made from four implants, with two straight and two 45 degrees distally tilted implants in an edentulous maxillary model. The results of the comparison showed that the digital technique was found to be the most trueness with a 177.50-μm linear deviation. The trueness of the open tray technique was 280 μm, and the closed tray technique showed the highest deviation with 885 μm. 4 A systematic review published by Papaspyridakos et al regarding conventional implant impressions stated that splinting significantly increases impression accuracy, especially in partial arch implant cases. 3 23

Finding the reference point for IOS is challenging for digital implant impressions of full arches. Alikhasi et al present higher discrepancies (177.50 µm) in edentulous maxilla full-arch with implant abutments for the IOS. The IOS measurement technique was a superposition of .stl data sets (best-fit alignment tool), and the IOS measurement level was .stl. Having these different levels (implant analog level or scan body level) where the files are merged and measured, a fair comparison between study results is hard to execute. This scanning strategy was different from the other select studies. 2 23 The authors started scanning from the palate's reference pin toward all scan bodies' right tuberosity and lingual surfaces. 4 Next, they scanned the scan bodies' buccal surfaces and occlusal surfaces. Accordingly, accuracy varies significantly with scan body type, IOS type, scanning strategy, and modification technique. One possible reason that this study, Alikhasi et al, showed more deviation was related to the scanning strategy.

Müller et al have investigated the effects of scanning protocols and confirmed that scanning accuracy differs depending on the protocol used. However, no one considered the effects of the rotation of the IOSs on their accuracy when performing full-arch scans. 30 Oh et al, in an *in vitro* study, assessed the effects of different scanning strategies on the accuracy of the scanned data. According to the authors, a segmental approach to scanning the region of interest first or a continuous scan method with the scanner head kept mostly in a horizontal position throughout the scanning can be used to obtain the full arch scan data. However, the rotation of IOS in the vertical direction should be minimized because it affects the accuracy of the stl. 34 Stefanelli et al, in an *in vitro* study, developed two scanning strategies for a full dental arch. However, the literature has no consolidated approach for a full arch in digital implant impression. 32

Similarly, Rech-Ortega et al compared the trueness of two techniques, a direct (or pick-up) technique with the elastomeric material polyether and a digital scanner (True Definition, 3M ESPE), by comparing measurements between implant replicas. Neither technique can be considered accurate in rehabilitations involving more than four implants. However, both techniques analyzed can be used with relative reliability, as the errors produced fell within the tolerance range established in the literature as acceptable (30–150 μm), 2 although it is advisable to make a verification splint before fabricating the definitive prosthesis. 2 Another reason for this linear and angular distortion presented in the studies is the methodology to assess the IOS's accuracy; the software works by making a comparison between the IOS . *stl* file and the reference scan's STL file using the best-fit algorithm function; this matching generates linear deviations between the two data sets that can be measured. 32

Chochlidakis et al, in the first clinical study, compared the trueness of digital and conventional maxillary implant impressions for fully edentulous patients. They concluded a positive correlation between implant number and 3D deviation. So, the angular deviation increases with the number of the implant. According to this study, a digital impression will be a challenge in full-arch cases. D'haese et al evaluated the accuracy of full-arch digital impressions compared with conventional impressions when performed on the abutment or implant level. In this study, the abutment level impressions were more accurate than implant level impressions because of the flat connection geometry, which provides a vertical stop. 35

Digital impressions for full-arch implant-supported prostheses can be as accurate as conventional impressions, depending on the IOS and software. 9 15 Previous *in vitro* studies investigating the effects of scanning protocols have also confirmed that scanning accuracy differs depending on the protocol used. 9 15 Kim et al 23 used 3D displacement of implant replicas and not those of scan bodies. The discrepancies are attributed to the inherent errors in converting the scan body position to the implant replica position using a digital library. Several commercial brands offer scan bodies for the different implant and IOS systems. Although there is adequate accuracy when comparing scan bodies' position in a virtual model, the algorithms in digital planning software still need development to position the implant replicas accurately.

Lastly, one study 26 showed that the True Definition Scanner might not achieve a clinically acceptable accuracy threshold as an exception. The clinician should be aware that Midmark purchased the True Definition trademark from 3M, but the new company did not report significant improvements in IOS. Digital scanning of full arches for implant-supported complete prostheses is promising and clinically acceptable; this systematic review ensures that IOS can be more accurate than conventional impression materials.

## Conclusion

Within the limitation of this study, it can be concluded that digital impressions using IOS present similar or better linear accuracy than conventional impression techniques. All the IOS included in this study presented acceptable accuracy (below the 200 µm threshold), except for the True Definition Scanner in one of the studies. The scan body lack of optimization might be attributed to divergence in the data.
