# Supplementary material for: Linear Accuracy of Intraoral Scanners for Full-Arch Impressions of Implant-Supported Prostheses: A Systematic Review and Meta-Analysis
Source: Eur J Dent. 2023 Jan 30;17(4):964–73. doi: 10.1055/s-0042-1758798 (PMC10756734; doi:10.1055/s-0042-1758798)
Supplement: Supplementary file 1 — Supplementary Material [file 10-1055-s-0042-1758798-s2272281.pdf]

Supplementary Table S1 Search strategy

Databases	Search strategy
PubMed<br>($n = 724$)	("Mouth, Edentulous"[MeSH Terms:noexp] OR "Jaw, Edentulous"[MeSH Terms:noexp] OR "Denture, Complete"[MeSH Terms:noexp] OR "full-arch"[Title/Abstract] OR "complete-arch"[Title/Abstract] OR Edentul*[Title/Abstract] OR "Complete denture"[Title/Abstract] OR "Complete dentures"[Title/Abstract]) AND ("digital"[Title/Abstract] OR "intraoral"[Title/Abstract] OR "intra oral"[Title/Abstract] OR "desktop"[Title/Abstract] OR "scanning"[Title/Abstract] OR "scanner"[Title/Abstract] OR "scanners"[Title/Abstract]) AND ("dental impression technique"[MeSH Terms] OR "dental impression technique"[Title/Abstract] OR "conventional"[Title/Abstract] OR "analog"[Title/Abstract] OR "alginate"[Title/Abstract] OR "polyether"[Title/Abstract] OR "polyvinylsiloxane"[Title/Abstract] OR "elastomers"[Title/Abstract] OR "elastomer"[Title/Abstract] OR "polysulfide"[Title/Abstract] OR "PVS"[Title/Abstract] OR impression*[Title/Abstract] OR "molding"[Title/Abstract])
Scopus<br>($n = 767$)	(INDEXTERMS ({Mouth, Edentulous} OR {Jaw, Edentulous} OR {Denture, Complete}) OR TITLE-ABS-KEY ({full-arch} OR {complete-arch} OR Edentul* OR {Complete denture} OR {Complete dentures})) AND (TITLE-ABS-KEY ({digital} OR {intraoral} OR {intra oral} OR {desktop} OR {scanning} OR {scanner} OR {scanners})) AND (INDEXTERMS ({dental impression technique}) OR TITLE-ABS-KEY ({dental impression technique} OR {conventional} OR {analog} OR {alginate} OR {polyether} OR {polyvinylsiloxane} OR {elastomers} OR {elastomer} OR {polysulfide} OR {PVS} OR impression* OR {molding}))
Web of Science<br>($n = 687$)	noft(("Mouth, Edentulous" OR "Jaw, Edentulous" OR "Denture, Complete" OR "full-arch" OR "complete-arch" OR Edentul* OR "Complete denture" OR "Complete dentures") AND ("digital" OR "intraoral" OR "intra oral" OR "desktop" OR "scanning" OR "scanner" OR "scanners") AND ("dental impression technique" OR "conventional" OR "analog" OR "alginate" OR "polyether" OR "polyvinylsiloxane" OR "elastomers" OR "elastomer" OR "polysulfide" OR "PVS" OR impression* OR "molding"))
Embase<br>($n = 631$)	('edentulism'/mj OR 'edentulous jaw'/mj OR 'complete denture,'/mj OR 'full-arch':ti,ab,kw OR 'complete-arch':ti,ab,kw OR Edentul*:ti,ab,kw OR 'Complete denture':ti,ab,kw OR 'Complete dentures':ti,ab,kw) AND ('digital':ti,ab,kw OR 'intraoral':ti,ab,kw OR 'intra oral':ti,ab,kw OR 'desktop':ti,ab,kw OR 'scanning':ti,ab,kw OR 'scanner':ti,ab,kw OR 'scanners':ti,ab,kw) AND ('dental impression'/mj OR 'dental impression':ti,ab,kw OR 'dental impression technique':ti,ab,kw OR 'conventional':ti,ab,kw OR 'analog':ti,ab,kw OR 'alginate':ti,ab,kw OR 'polyether':ti,ab,kw OR 'polyvinylsiloxane':ti,ab,kw OR 'elastomers':ti,ab,kw OR 'elastomer':ti,ab,kw OR 'polysulfide':ti,ab,kw OR 'PVS':ti,ab,kw OR impression*:ti,ab,kw OR 'molding':ti,ab,kw)
Cochrane Library<br>($n = 122$)	#1 MeSH descriptor: [Mouth, Edentulous] this term only; OR #2 MeSH descriptor: [Jaw, Edentulous] this term only; OR #3 MeSH descriptor: [Denture, Complete] this term only; OR #4 ("full-arch" OR "complete-arch" OR Edentul* OR "Complete denture" OR "Complete dentures"):ti,ab,kw<br>#1 OR #2 OR #3 OR #4 = #5<br>AND #6("digital" OR "intraoral" OR "intra oral" OR "desktop" OR "scanning" OR "scanner" OR "scanners"):ti,ab,kw<br>AND #7 MeSH descriptor: [Dental Impression Technique] this term only; OR #8 ("dental impression technique" OR "conventional" OR "analog" OR "alginate" OR "polyether" OR "polyvinylsiloxane" OR "elastomers" OR "elastomer" OR "polysulfide" OR "PVS" OR impression* OR "molding"):ti,ab,kw<br>#7 OR #8 = #9<br>#5 AND #6 AND #9
LILACS<br>($n = 36$)	(mh:("mouth, edentulous" OR "jaw, edentulous" OR "denture, complete") OR tw:("full-arch" OR "complete-arch" OR edentul* OR "complete denture" OR "complete dentures")) AND (tw: ("digital" OR "intraoral" OR "intra oral" OR "desktop" OR "scanning" OR "scanner" OR "scanners")) AND (mh:("dental impression technique") OR tw:("dental impression technique" OR "conventional" OR "analog" OR "alginate" OR "polyether" OR "polyvinylsiloxane" OR "elastomers" OR "elastomer" OR "polysulfide" OR "pvs" OR impression* OR "molding")) AND (db:("LILACS"))
Clinical Trials<br>($n = 14$)	("digital" OR "intraoral" OR "intra oral" OR "desktop" OR "scanning" OR "scanner" OR "scanners") AND ("dental impression technique" OR "dental impression technique" OR "conventional" OR "analog" OR "impression" OR "molding")
Easy Dans<br>(Open Gray)<br>($n = 56$)	("digital" OR "intraoral" OR "intra oral" OR "desktop" OR "scanning" OR "scanner" OR "scanners") AND ("dental impression technique" OR "dental impression technique" OR "conventional" OR "analog" OR "impression" OR "molding")
ProQuest (Dissertation and Theses)<br>($n = 43$)	noft(("Mouth, Edentulous" OR "Jaw, Edentulous" OR "Denture, Complete" OR "full-arch" OR "complete-arch" OR Edentul* OR "Complete denture" OR "Complete dentures") AND ("digital" OR "intraoral" OR "intra oral" OR "desktop" OR "scanning" OR "scanner" OR "scanners") AND ("dental impression technique" OR "conventional" OR "analog" OR "alginate" OR "polyether" OR "polyvinylsiloxane" OR "elastomers" OR "elastomer" OR "polysulfide" OR "PVS" OR impression* OR "molding"))
Google Scholar<br>($n = 100$)	("digital" OR "intraoral" OR "intra oral" OR "desktop" OR "scanning" OR "scanner" OR "scanners") AND ("dental impression technique" OR "dental impression technique" OR "conventional" OR "analog" OR "impression" OR "molding")

 © 2023. The Author(s).

Supplementary Table S2 Exclusion reason

First author, Year	Article	Reason
Ender, 2011	Full arch scans: Conventional versus digital impressions – An in vitro study	Do not evaluate conventional and digital in implant impression
Yuzbasioglu, 2014	Comparison of digital and conventional impression techniques: evaluation of patients' perception, treatment comfort, effectiveness and clinical outcomes	Not show measurements
Ender, 2015	In vitro evaluation of the accuracy of conventional and digital methods of obtaining full-arch dental impressions	Do not evaluate conventional and digital in implant impression
Rhee, 2015	Comparison of intraoral scanning and conventional impression techniques using 3-dimensional superimposition	Not compare analog and digital
Nedelcu, 2017	Accuracy and precision of 3 intraoral scanners and accuracy of conventional impressions: a novel in vivo analysis method	Not analyze full arch, only the teeth separately
Basaki, 2017	Accuracy of Digital vs. Conventional Implant Impression Approach: A Three-Dimensional Comparative In Vitro Analysis	Partial Arch
Malik, 2018	Comparison of Accuracy Between a Conventional and Two Digital Intraoral Impression Techniques	Do not evaluate conventional and digital in implant impression
Nedelcu, 2018	Accuracy and precision of 3 intraoral scanners and accuracy of conventional impressions: A novel in vivo analysis method	In vivo analysis method
Cappare, 2019	Conventional versus Digital Impressions for Full Arch Screw-Retained Maxillary Rehabilitations: A Randomized Clinical Trial	Analog and digital fabrication of full-arch rehabilitation
Lo russo, 2019	Three-dimensional differences between intraoral scans and conventional impressions of edentulous jaws: A clinical study	Not compare analog and digital
Alsharbaty, 2020	A Clinical Comparative Study of 3-Dimensional Accuracy between Digital and Conventional Implant Impression Techniques	Partial Arch
Chochlidakis, 2020	Digital Versus Conventional Full-arch Implant Impressions: A prospective study on 16 edentulous Maxillae	Clinical Study
Schmidt, 20220	Accuracy of Digital and Conventional Full-Arch Impressions in Patients: An Update	Do not evaluate conventional and digital in implant impression
Rani D'haese, 2021	In Vitro Accuracy of Digital and Conventional Impressions for Full-Arch Implant-Supported Prostheses	Evaluate the abutment and implant level.
Léon, 2021	Comparison of conventional, photogrammetry, and intraoral scanning accuracy of complete-arch implant impression procedures evaluated with a coordinate measuring machine	Photogrammetry
Elawady, 2022	Clinical influence of digital vs analog impressions in all-on-4 implant prostheses: a randomized controlled trial	Analog and digital fabrication of full-arch rehabilitation
Farhan, 2021	Comparison of the accuracy of intraoral digital impression system and conventional impression techniques for multiple implants in the full-arch edentulous mandible	Not show measurements
Jorquera,2021	Evaluation of trueness and precision of two intraoral scanners and a conventional impression: an in vivo clinical study	Do not evaluate conventional and digital in implant impression
Li, 2021	Accuracy of impression-making methods in edentulous arches: An in vitro study encompassing conventional and digital methods	Do not evaluate conventional and digital in implant impression
Sallorenzo, 2021	Comparative study of the accuracy of an implant intraoral scanner and that of a conventional intraoral scanner for complete-arch fixed dental prostheses	Do not evaluate conventional and digital in implant impression
Schimmel, 2021	Accuracy of intraoral scanning in completely and partially edentulous maxillary and mandibular jaws: an in vitro analysis	Do not evaluate conventional and digital in implant impression
Seo, 2021	A new method to evaluate trueness and precision of digital and conventional impression techniques for complete dental arch	Do not evaluate conventional and digital in implant impression

 © 2023. The Author(s).
